# Are Safe Sleep Practice Recommendations For Infants Being Applied Among Caregivers?

**DOI:** 10.7759/cureus.12133

**Published:** 2020-12-17

**Authors:** Turki S Alahmadi, Mrouge Sobaihi, Maysaa A Banjari, Kholoud Mohammed A Bakheet, Sara Ali Modan Alghamdi, Adel S Alharbi

**Affiliations:** 1 Pediatric Department, Faculty of Medicine, King Abdulaziz University, Jeddah, SAU; 2 Pediatric Department, Faculty of Medicine, King Abdulaziz University, Rabigh, SAU; 3 Pediatric Department, King Faisal Specialist Hospital and Research Centre, Jeddah, SAU; 4 Pediatric Department, Prince Sultan Military Medical City, Riyadh, SAU

**Keywords:** safe sleep, sleep position, infant sleep, sudden infant death syndrome (sids)

## Abstract

Background

Sudden infant death syndrome (SIDS) is defined as the sudden unexpected death of an infant, even after investigations and autopsy. SIDS is related to many factors, such as the baby’s position and objects in the crib. Adherence to safe sleep recommendations in Saudi Arabia is unclear. This study aims to assess caregivers’ implementation of safe sleep practices and if they received any safe sleep education through health care workers.

Methods

This was a cross-sectional, descriptive study. Inclusion criteria included all infants below the age of one year. Exclusion criteria included infants who were born premature, used ventilation, had a tracheostomy, any anomaly in the upper airway, or underwent spine surgery. A semi-structured questionnaire was used. Data were collected from mothers who had infants visiting the outpatient department of King Abdulaziz University Hospital in Jeddah, Saudi Arabia. An electronic survey was also created and published on a social platform.

Statistical analysis was conducted with the aid of the Statistical Package for Social Sciences (SPSS) software, version 26 (IBM SPSS Statistics, Armonk, NY).

Results

Among 506 participants, only 22.5% were found to receive education about safe practices from health care providers. Fortunately, most of the infants (63.2%) were found to sleep in a supine position most of the nights. Adherent caregivers to placing the child in a designated baby bed and in a supine position most nights represented 44.86% of the sample. However, when asked about placing any of the following objects in the bed (pillows, blankets, soft toys, hard toys, and electric wires), the percentage of adherence dropped down to only 1.58%.

Conclusion

There was an obvious non-adherence among caregivers and a possible lack of knowledge of safe sleep recommendations for infants. This highlights the need for optimal education by health care workers and the rule of media and campaigns is obvious and essential to improving their practices and, hopefully, decreasing the risk of SIDS.

## Introduction

Sudden unexpected infant death (SUID) is a devastating event. As suggested by the name, it describes the death of a child less than one year of age for any reason whether identified or unidentified [1]. After a thorough case investigation, SUID can be attributed to entrapment, suffocation, infections, trauma, and diseases of metabolism. If no specific cause of death is established, the term “Sudden Infant Death Syndrome” (SIDS) is used [2].

According to the Centers for Disease Control and Prevention (CDC), during the year 2016, the deaths of 1,500 infants were solely attributed to SIDS in the United States [3].

Awareness of some of the mentioned causes has led to the development of guidelines worldwide in an attempt to reduce the incidence of sleep-related infant deaths. These guidelines focus on giving anticipatory guidance to parents and caregivers about safe sleep practices. The American Academy of Pediatrics (AAP) released important recommendations to prevent the occurrence of SIDS that includes using a firm-sleep surface, a pacifier, breastfeeding, placing the infant in a supine position, and avoidance of smoke exposure [4].

Despite the availability of strong guidelines, it was noticed that many parents report either being non-adherent to or unaware of safe sleep recommendations. A Malaysian study that was conducted on the parents of preterm infants found that a high percentage of parents practiced bed-sharing and positioned the infant in non-supine positions, in addition to having a high rate of smoking in the household [5]. A similar study that was conducted in Turkey revealed that a side-sleeping position was preferred among the parents, in addition to using a soft-sleep surface [6].

Moreover, in a case-control study conducted in Lithuania, significant factors for SIDS included bed-sharing with parents and maternal or paternal smoking during pregnancy [7].

In Saudi Arabia, no studies are investigating the number of deaths related to SIDS. Also, it is unclear as to what the degree of lack of adherence to safe sleep practices is and what the contributing factors are. We hypothesize that some cultural practices, parental fatigue, convenience, as well as other factors, may play a role.

Through our study, we would like to answer the following questions:

1) Are safe sleep practices being applied among caregivers?

2) Did they receive anticipatory guidance on safe sleep before discharge from health care providers?

We anticipate that the results of our study will help to increase awareness about healthy sleep practices among caregivers. In addition, we hope that the identified barriers will help in creating solutions for overcoming them. The study results will also guide education campaigns about safe sleep and encourage health care providers to deliver guidance about safe sleep practices during clinical encounters, as they conveniently interact with most new parents and can educate them easily [8].

With time, this is expected to increase overall adherence to safe sleep practices and hopefully contribute to decreasing the incidence of sleep-related deaths in infants.

## Materials and methods

The study was officially reviewed and approved by the Unit of Biomedical Ethics, Faculty of Medicine, King Abdulaziz University, Jeddah, Saudi Arabia (approval HA-02-J-008). Informed consent was taken from all caregivers of participants.

This was a cross-sectional, descriptive study. Exclusion criteria included infants who were born premature, used ventilation, had a tracheostomy, any anomaly in the upper airway, or underwent spine surgery. Data were collected from mothers who had children below the age of one-year-old visiting the outpatient department of the King Abdulaziz University Hospital. Also, an electronic survey was created and published on a social platform.

The study used a semi-structured questionnaire (see Appendix). It was prepared by the researchers based on the literature review conducted at the start of the study. There was a total of 44 questions, 18 of which asked about parents’ and children’s sociodemographic characteristics, while the remaining questions were related to sleep habits applied by the caregiver. The questions about sleep habits were decided based on the known risk factors for developing SIDS [4].

Statistical analysis was conducted with the aid of the Statistical Package for Social Sciences (SPSS) software (IBM SPSS Statistics, Armonk, NY).

## Results

A total of 520 patients agreed to participate in this study. Fourteen of them were excluded due to missing data. The total number of infants studied were 506 infants.

Demographics of the infants

**Figure 1:**
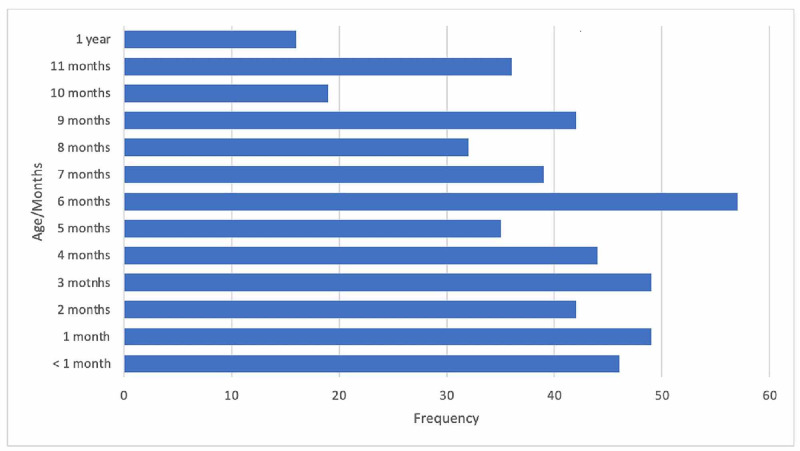
Percentage of 506 infants ages (in months)

The median age of infants was five months, and the age distribution ranged between one day to 360 days. Of these, 322 (63.3%) were aged six months or younger. Figure 1 demonstrates the frequencies of each age. Furthermore, 272 (53.8%) of the sample were males. Further details are described in Table 1.

**Table 1:** Demographics of the Sample (n = 506)

Demographics	N (%)
Gender	
Male	272 (53.8)
Female	234 (46.2)
Total number of children in the household	
One child	185 (36.6)
Two to three	197 (38.9)
Four to five	94 (18.6)
More than five	30 (5.9)
Does the child have any chronic medical illnesses?	
Yes	35 (6.9)
No	471 (93.1)
Mother’s education	
High school or lower	123 (24.3)
Postgraduate studies	383 (75.7)
Father’s education	
High school or lower	115 (22.7)
Postgraduate studies	391 (77.3)
Mother’s employment	
No	340 (67.2)
Yes	166 (32.8)
Father’s employment	
No	10 (2)
Yes	496 (98)

Demographics of the parents

The mothers’ ages ranged between 18 - 47 years old with a median of 29, while the fathers’ ages ranged between 24 - 60 years old with a median of 34. As for the parents’ education, postgraduate studies were more prevalent in both the mothers and fathers (75.7% and 77.3%, respectively). Further details are noted in Table 1.

At the time of conducting this study, it was noticed that around 17% of the mothers admitted that they used tobacco products. Infants, six months old and younger, were noted to sleep mostly in the same room as their parents (91%). This practice also persisted among infants who were seven to 12 months of age (89.7%). Moreover, infants were found to sleep in their designated baby bed most of the nights in 69.8% of the sample; 33.8% of the mothers admitted that they shared their adult beds with their infants. More details are present in Table 2. The supine position was the preferred position for most of the nights in 63.2% of the sample. However, sleeping on the baby’s side or in the prone position most nights were also noted to occur, but to a lesser degree, in 37% and 22.1%, respectively (Table 3).

**Table 2:** Characteristics of the Child’s Household Environment and His\Her Sleep Habits

Risk Factors of Child’s Household Environment and Sleep Habits	N (%)
Presence of smokers at home	188 (37.2)
Mother smoked during pregnancy	85 (16.8)
Parents were educated about safe sleep practices by a health care professional	114 (22.5)
Where does the child sleep? (6 months or younger)	
He/she sleeps alone	22 (6.8)
With the parents	293 (91)
With other children	7 (2.2)
Where does the child sleep? (7 months to 12 months)	
He/she sleeps alone	11 (6)
With the parents	165 (89.7)
With other children	8 (4.3)
Pacifier use:	
Always	118 (23.3)
Sometimes	140 (27.7)
Rarely	248 (49)
Swaddling	
Always	158 (31.2)
Sometimes	159 (31.4)
Rarely	189 (37.4)
Mattress surface:	
Soft	177 (35)
Firm	328 (64.9
Child is currently breastfeeding	
Less than 6 months	255 (79.1)
6 months – 12 months	135 (73.3)

**Table 3:** Characteristics of the Child’s Sleeping Environment and Habits

Child’s Sleeping Environment and Habits				
	Always	Sometimes	Rare	Never
Sleep location				
Designated baby bed	353 (69.8)	52 (10.3)	31 (6.1)	70 (13.8)
Adult bed (alone)	46 (9.1)	78 (15.4)	30 (5.9)	352 (69.6)
Adult bed with parents	171 (33.8)	149 (29.4)	44 (8.7)	142 (28.1)
Ground	34 (6.7)	137 (27.1)	66 (13.0)	269 (53.2)
Car seat	45 (8.9)	130 (25.7)	81 (16.0)	250 (49.4)
Couch	21 (4.2)	126 (24.9)	79 (15.6)	280 (55.3)
Swing	34 (6.7)	105 (20.8)	55 (10.9)	312 (61.7)
Child carrier	26 (5.1)	63 (12.5)	28 (5.5)	389 (76.9)
Child sleep position				
On his\her back	320 (63.2)	127 (25.1)	20 (4.0)	39 (7.7)
On his\her side	187 (37.0)	151 (29.8)	62 (12.3)	106 (20.9)
On his\her abdomen	112 (22.1)	135 (26.7)	48 (9.5)	211 (41.7)
Presence of objects in the bed:				
Pillow	334 (66.0)	45 (8.9)	18 (3.6)	109 (21.5)
Blanket	387 (76.5)	55 (9.9)	21 (4.2)	48 (9.5)
Soft toys	139 (27.5)	76 (15.0)	34 (6.7)	257 (50.8)
Hard toys	21 (4.2)	39 (7.7)	28 (5.5)	418 (82.6)
Mobile wires	26 (5.1)	31 (6.1)	27 (5.3)	422 (83.4)

## Discussion

The present study revealed the implementation of safe sleep recommendations for infants by their caregivers was suboptimal.

Child’s sleeping arrangements

Infants who share rooms with their parents while being placed in designated baby beds have less likelihood of developing SIDS [4, 9-10]. In this study, infants were sleeping in their parents’ rooms 90.5% of the time; this observation did not differ significantly in infants less than six months of age (91%) vs infants six to 12 months of age (89.7%). Most children had their own designated crib; however, 13.8% never had a crib. What was obvious in this study was that there were many breaches of what is considered as a safe sleep placement for infants. Infants were placed to sleep in adult beds with parents, a couch, a car seat, a child carrier, or a swing frequently.

In a study done in Sweden, only 50.9% of infants below three months slept in their own cribs [11]. The percentage rose to 57.9% in those who were three to six months old, while another study in the United States showed a similar percentage of 48.7%. Our study had a higher percentage of 69.8%, but it is still considered somewhat low considering that it is the optimal location for an infant’s sleep [12]. In order to reduce the incidence of SIDS, education about the risks of bed-sharing should be addressed adequately. 

Child’s sleeping position

Sleeping in the supine position is known to be the most iconic change since the introduction of the Back to Sleep campaign because it confers the lowest risk of SIDS [12-14]. In an analysis of SIDS and related deaths, a prone position was noted in 66% of the sample, and an adult bed was the most common location for last sleep [15]. Moreover, in a European case-control study, 62% of SIDS deaths were attributable to being in a prone position or getting the head covered [16]. Mechanisms of SIDS in this setting include airway obstruction, rebreathing CO_2_, positional cerebral hypoxia, and overheating [17].

In a study conducted in Japan during the one-month checkup of infants, 96.7% of them were exclusively placed in the supine position [18], while another study done on South Dakota mothers of infants younger than six months showed a percentage of 87.6% [19]. Moreover, a study in Turkey in 2018 revealed shocking results of infants being placed in a supine position in only 22.1% of the sample and 27.2% of them in a combination of two or three positions [6].

In this study, infants were placed on their back 63.2% of the time. When analyzed, the percentage for infants less than six months rose to 68.9% (222/322), while 53.2% (98/184) of infants older than six months were placed on their backs. This is clearly a concerning number and must be emphasized through adequate teachings on safe sleep habits. It is worth noting that infants younger than six months did not acquire the milestone of turning to roll over, making them more at risk for suffocation during sleep. 

Some characteristics of the child’s household environment and sleep habits

In one of the recent and largest case-control studies of SIDS, breastfeeding was found to decrease the risk of SIDS by 50% at all ages [20]. In the current study, breastfeeding was documented in 79.1% of infants below six months of age; however, mothers who breastfed their infants were more likely to place them in a prone position (p < 0.05).

Furthermore, breastfed infants were more likely to share the bed with their parents (80.9% vs 19.1%). This is similar to what was found in other studies [21-22]. This can be explained by the mothers' tendency to practice bed-sharing with their infants in order to decrease sleep disturbances for the parents and the child [11, 21].

Maternal smoking during pregnancy was found to be related to the impairment of arousal processes of infants, which could increase SIDS risk [23]. Unfortunately, In the current study, around 17% of the mothers were smokers during pregnancy. Moreover, even though postnatal smoking is an important risk factor for SIDS [24], it was noted that there were household members who smoked in the child’s environment among 37.2% of the sample.

Education on safe sleep practices

Educating mothers early after delivery on safe sleep practices has a significant effect on the development of positive and autonomous sleep habits in infants [25]. In a study done in the United States, many mothers admitted that they did not understand the rationale behind sleep recommendations and noted many misconceptions, like pillows and soft blanket use to help cushion the baby [26]. In this study, only 22.5% were found to have received education from health care professionals about safe sleep practices for their infants. On further analysis, those who didn’t receive education were more likely to follow safe sleep practices compared to those who received an education. This is unexpected and can point to a defect in the process of education. This emphasizes the importance of educating parents while accounting for their different educational backgrounds, current knowledge, and misconceptions. 

Adherence level to safe sleep practices

**Figure 2:**
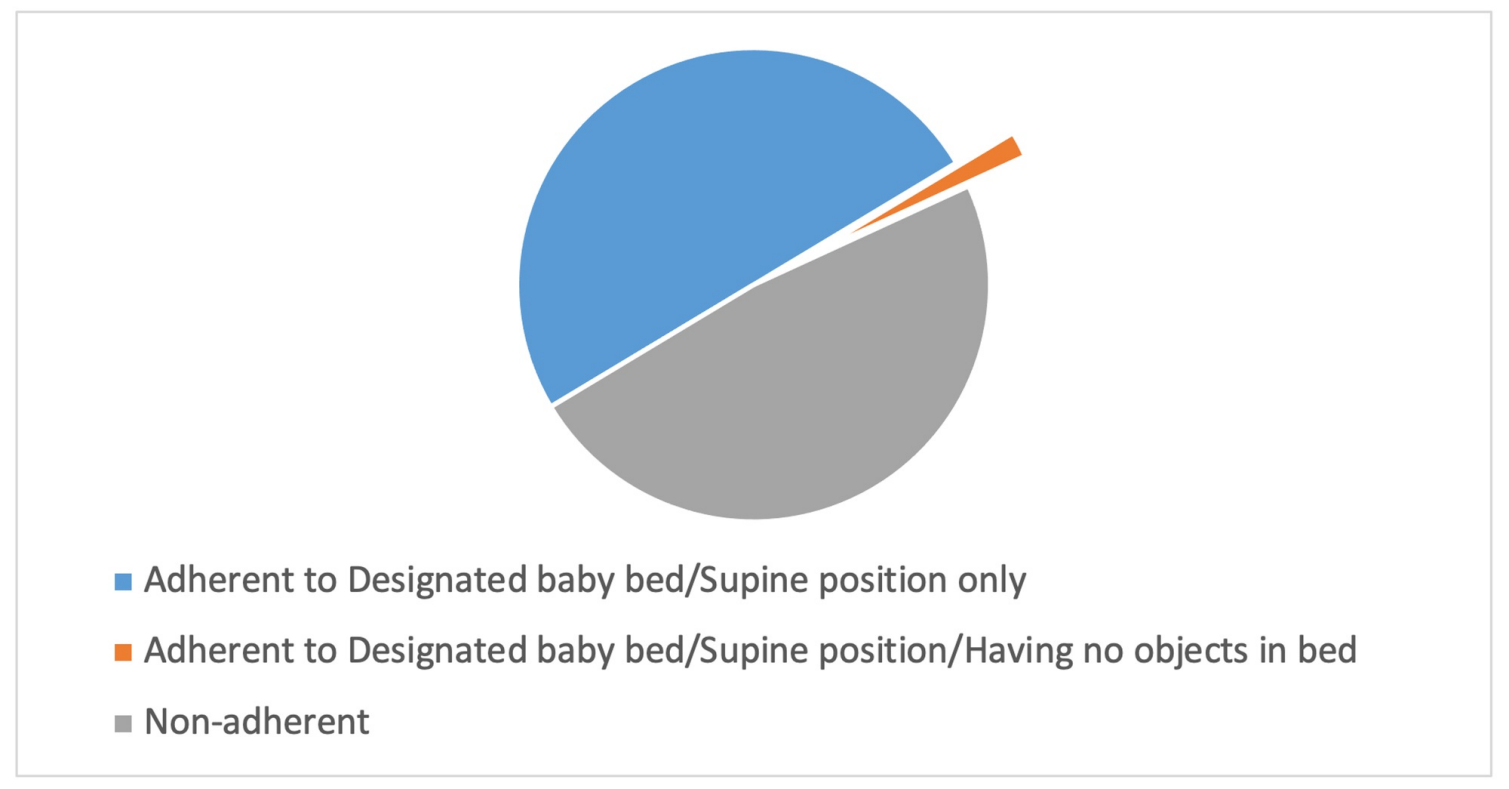
Level of adherence to safe sleep practices among the sample

Caregivers were labeled “adherent” if they implemented the following recommendations: placing the child in a supine position in a designated baby bed most of the nights and the bed has no objects, such as pillows, blankets, toys, or electric wires. These were specified based on the recommendations by the AAP [4]. Adherent caregivers to placing the child in a designated baby bed and in a supine position most nights represented 44.86% of the sample. However, when asked about placing any of the following objects in the bed (pillows, blankets, soft toys, hard toys, and electric wires), the percentage of adherence dropped down to only 1.58% (Figure 2).

Limitations of this study

There are a number of limitations to this study. First, the parents’ knowledge was not assessed, which could help to facilitate educational campaigns and point exactly towards the defects that need to be addressed. Also, recall bias is an important factor to consider, as many of the questions depend on memory. Parents are usually stressed and exhausted during the first year of an infant’s life, which could have affected their answers. Conducting a qualitative study and interviewing the parents using open-ended questions can help eliminate any misunderstandings and shed light on their misconceptions. Also, future studies should be conducted in an interventional manner to test the effectiveness of education measures used, as well as to assess parents’ comprehension and information retention.

## Conclusions

Although more than 75% of the caregivers in this study were of postgraduate level, there was obvious nonadherence to safe sleep practices for their infants in 98.42% of the sample. The need for optimal education by health care workers after delivery and in routine visits, in addition to the utilization of media and campaigns in public places, is obvious and essential as it can vastly improve their knowledge and adherence, ultimately decreasing the risk of SIDS among the population.

## Appendices

Are safe sleep practice recommendations for infants being applied among caregivers?

Child’s information:

- Child’s name: …………………………………….

- Gender:

o   Female

o   Male

- Date of birth:    /      /

- History of chronic illnesses (asthma, cerebral palsy, congenital heart disease, etc.):

 ………………………………………………………………………

- Medications used chronically:

 ………………………………………………………………………

-Was the child born prior to completing 37 weeks of pregnancy?

o   Yes

o   No

-Did the child need any method of assisted ventilation during his life? {intubation, bilevel positive airway pressure (BIPAP), continuous positive airway pressure (CPAP)}

o   Yes

o   No

-Does the child have a tracheostomy?

o   Yes

o   No

-Did the child undergo any surgery of the spinal cord?

o   Yes

o   No

-Does the child have any narrowing/disease in the upper airway?

o   Yes

o   No

-Is the child breastfed?

o   Yes

o   No

o   Sometimes

_______________________________________________________________

Parents’ information

-Mother’s age: …………………………………….

-Father’s age: …………………………………….

-Mother’s education level: …………………………………….

-Father’s education level: …………………………………….

-Mother’s job: …………………………………….

-Father’s job: …………………………………….

-Any smokers in the household?

o   Yes

o   No

-Did the mother smoke during pregnancy?

o   Yes

o   No

-Number of children at home: …………………………………….

-Number of rooms at home: …………………………………….

_______________________________________________________________

Child’s sleep pattern and environment

-Does the child sleep:

o   Alone

o   With parents

o   With other children

-Where does the child sleep usually?

o   Designated baby bed

o   Adult bed with parents

o   Adult bed alone

o   Ground

o   Car seat

o   Couch

o   Swing

o   Child carrier

-What is the child’s position during sleep?

o   On his back

o   On his side

o   On his abdomen

-Does the child use pacifier during sleep?

o   Sometimes

o   Always

o   Rarely

-Does the child require swaddling so he can sleep?

o   Sometimes

o   Always

o   Rarely

-How common do these items exist in the child’s crib/next to him while he sleeps?

o   Pillow

o   Blanket

o   Soft toys

o   Hard toys

o   Mobile wires

-How is the bed mattress?

o   Soft

o   Firm

- Were the family members educated about safe sleep practices by a health care professional?

o   Yes

o   No
